# Cultivating awareness: How botanical gardens can foster public engagement with plant pathogens

**DOI:** 10.1371/journal.ppat.1013629

**Published:** 2025-10-28

**Authors:** Dagmar Renate Hann, Gudrun Kadereit

**Affiliations:** 1 Faculty of Biology, Genetics, Ludwig-Maximilians-University Munich, München, Germany; 2 Faculty of Biology, Systematics, Biodiversity and Evolution of Plants, Ludwig-Maximilians-University Munich, München, Germany; 3 Natural History Collections of Bavaria, Botanical Garden Munich, München, Germany; University of Tübingen: Eberhard Karls Universitat Tubingen, GERMANY

## Abstract

Phytopathogens are a growing global threat to food security, economies, and ecosystems, yet public awareness and policy support often lag behind scientific innovations. Despite widespread pesticide use, 20–40% of global crop yields are lost to pests and diseases (FAO). Biotechnology and novel breeding strategies provide powerful tools to counter these threats, but their deployment hinges on public trust. Here, we review the societal risks posed by phytopathogens and cultural differences in public acceptance and regulatory frameworks. We discuss key challenges in plant science communication and the importance of trusted role-model communicators, including a concrete example where science-led storytelling and participatory engagement have accelerated adoption. We further propose that Botanical Gardens can act as scalable and adoptable platforms for plant health science communication to facilitate the translation into real-world applications.

## 1. How do phytopathogens impact society?

Phytopathogens remain a persistent and evolving threat to local and global agriculture [1]. The reduction in yields can lead to food price volatility and the loss of livelihood in subsistence farming. For example, the quarantine bacterium *Xylella fastidiosa,* for which no treatment currently exists, was first identified in Apulia in Europe in 2013 (Table 1). This region produces around 30% of Italy's olive oil and is home to culturally important, century-old trees. While the European Commission imposed strict phytosanitary measures, economic, cultural, and social concerns fueled resistance to containment efforts. This led to a widespread, continuing loss of olive trees and severe economic damage [2]. Resistant cultivars could be a solution but breeding or cultivation of those takes time. Also, resistance breeding in clonal monocultures, such as the Cavendish banana, is challenging. Thus the emergence of *Fusarium oxysporum* f. sp. *cubense* TR4 remains a threat to global Cavendish banana production [3].

**Table 1:** Selection of emerging phytopathogens and der global prevelance/ economic impact.

Pathogen and key strain(s)	Major host(s) and global prevalence/economic impact	Risk category
*Xylella fastidiosa*	Olive trees in southern Italy	Emerging pathogen, no effective treatment, only phytosanitary measures available
*Zymoseptoria tritici*	Major wheat disease with up to 40% yield loss	Rapidly increasing fungicide resistance
*Fusarium oxysporum* f. sp. *cubense* TR4	Cavendish banana	No genetic diversity due to clonal propagation

But also increasing pesticide resistances in the absence of the discovery of novel treatments challenge agriculture. For example, resistance of the wheat pathogen *Zymoseptoria tritici* to Demethylation inhibitors, one of the most widely applied fungicides, is rapidly increasing [4]. Biotechnology could provide a solution [5,6], such as for the resistance engineering of the Rainbow papaya that has provided a sustainable and effective control against the Papaya ringspot virus [7]. Yet, their success often hinges not just on technical merit but also on societal acceptance and regulatory frameworks [8]. Effective science communication can act as a bridge between laboratory discoveries and real-world implementation. Without it, even well-validated innovations risk rejection, as demonstrated by the public resistance to genetically modified (GM) crops in many regions including Germany [9].

## 2. Cultural differences in public acceptance and regulatory frameworks?

Over the past 25 years, global biotech crop production doubled to around 190 million hectares being cultivated in 2021 [8]. Slightly less than half of these were grown in industrialized countries, primarily the United States, Canada, and Australia. Of these, the United States has led the development and adoption of GM crops, accounting for up to 30% of global production. The remainder were produced in developing nations, notably Brazil, Argentina and India [8]. While some countries have minimal legislative barriers for biotech crops, others enforce strict process-oriented legislation. Canada, the only country with product-based legislation, produces around 7% of global GM crops. In the European Union, only Spain and Portugal cultivate GM crops. Consumer acceptance and regulatory frameworks strongly correlate [9]. In Europe, where public trust in GM crops is rather low, regulatory frameworks are highly restrictive (EC 1829/2003), while in the US a higher public acceptance is reflected in permissive regulations (2017—Coordinated Framework for the Regulation of Biotechnology). These global patterns illustrate how adoption is not only technical but also cultural.

In alignment with the “cultural theory,” these regional and cultural differences might not only be based on scientific understanding but also cultural values, personal beliefs, and historical experiences with science and governance [10]. For instance, a farmer's willingness to adopt GM crops may be influenced by community norms, perceived naturalness, and trust in the institutions promoting the technology. Trust, particularly in scientists, regulators, and industry, strongly predicts support for agricultural biotechnologies [10]. Early stakeholder engagement through participatory approaches can enhance ownership, reduce resistance, and support informed decision-making.

## 3. What challenges hinder effective communication in plant science?

Biotechnological advances face skepticism in the plant sector but are more accepted in human health. This contrast may be partly explained by plant awareness disparity (PAD) [11]. According to PAD, plant science receives less attention in media, classrooms, and textbooks. In consequence, many people are less aware of plant disease impacts on agriculture.

Despite broad agreement on the importance of science communication in this field, many research programs lack a comprehensive strategy. Most scientists receive little formal training in communication, public engagement, and risk perception [12]. Many efforts rely on the assumption that increasing scientific literacy will lead to acceptance, a deficit model that often fails to connect with audience values and concerns [13]. In plant science, PAD and persistent misinformation regarding GM crops in Europe underscore the consequences of weak communication [14]. To summarize, discussion about biotechnologies often evokes ethical, ecological, or political concerns that require nuanced and empathetic dialogue—something traditional outreach rarely achieves.

## 4. What strategies have proven effective in science communication?

Narrative storytelling makes topics more relatable [15], especially when it comes from role models with scientific prominence. Take Sir David Attenborough with his landmark BBC series “The private life of Plants” (1995) and most recently “The green planet” (BBC, 2022), who profoundly impacted plant science communication through engaging storytelling and stunning visuals [16]. Also, scientists such as Pamela Ronald made an impact. In her 2015 TED talk “The case for engineering our food” she reached more than two million listeners (https://www.ted.com/talks/pamela_ronald_the_case_for_engineering_our_food).

Public forums, citizen science, and deliberative workshops build trust through dialogue between researchers and communities [17]. Transdisciplinary collaborations further enhance reach and resonance and partnering with local extension workers, community leaders, and NGOs can ensure that messages are grounded in local realities and communicated by trusted messengers [18]. Community leaders and local realities were corroborated during the development of the virus-resistant Rainbow papaya [7]. The Hawaiian papaya industry was close to collapse due to the papaya ringspot virus. Treatments were unavailable, but genetic engineering offered a solution. Researchers actively collaborated with Hawaiian farmers, addressed their concerns and demonstrated clear economic benefits. This open dialogue resulted in broad acceptance of the Rainbow papaya [19].

## 5. How Botanical Gardens can serve as platforms for science communication: A blueprint

**Fig 1:**
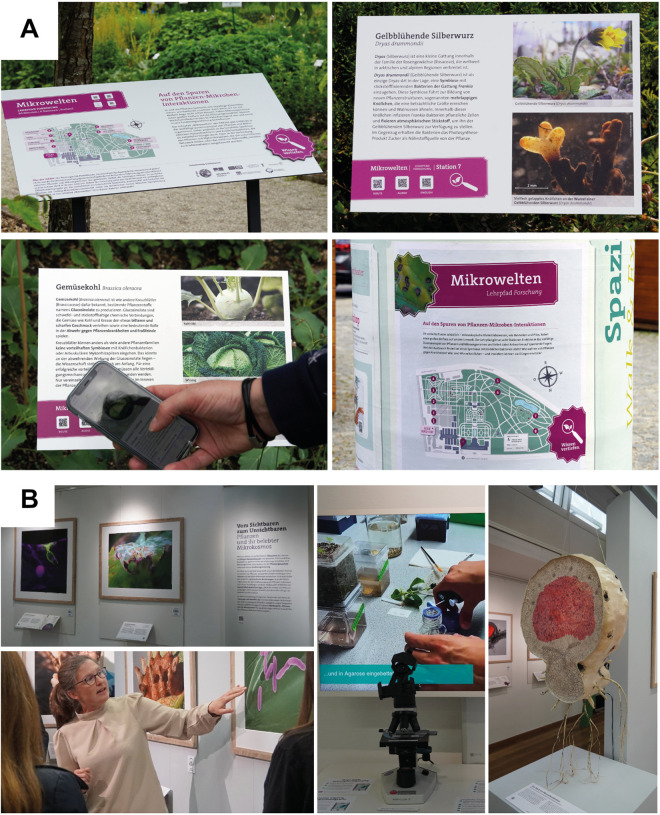
TRR356 Outreach activities in the Botanical Garden Munich-Nymphenburg. **A)** Teaching path through the living collection of the Botanical Garden Munich-Nymphenburg. **B)** Imressions of plant–microbe exhibitions at the Botanical Garden Munich-Nymphenburg. The image on the top shows a range of exhibits while the one on the bottom left shows a guided tour through the exhibition with macro- and microscopic images displayed in the back, on the left a digital media and experimental station is show as well as a giant model of a root nodule by the artist Alexandra Hendrikoff.

Botanical Gardens are neutral, trusted spaces annually attracting millions of visitors worldwide. Transregional networks and collaborations across countries, such as the International Plant Exchange Network (IPEN), offer unique opportunities for outreach that transcends local boundaries [20]. As living classrooms, Botanical Gardens make plant-microbe research tangible and relevant to diverse audiences by combining scientific exhibits, hands-on demonstrations, and citizen science initiatives. For example, the outreach project of the DFG-funded TRR356 *PlantMicrobe* involves the Botanical Garden Munich-Nymphenburg, that attracts more than 350,000 visitors and 500 school classes yearly, to engage the public in plant-microbe interactions. A permanent plant–microbe teaching path through the Gardens, living with infographics and audios, invites visitors to think and reflect about these invisible interactors and can provide a great resource for dialogue, especially in combination with guided tours (Fig 1A). The modular nature of such teaching paths makes them transferable to other Gardens. Yearly, modular multi-media exhibitions featuring different aspects of plant–microbe interactions attract several thousand visitors (Fig 1B). The 2024 exhibition was continued in the Botanical Garden Bochum, demonstrating the transferability of such exhibitions. Exhibitions and infographics can be used for the dissemination of digital formats, such as audios, videos, animations, and podcasts. For example, the bi-weekly TRR356 plant–microbe podcast (https://trr356plantmicrobe.de/public-outreach/podcast/index.html), is connected to both the teaching path and the exhibition elements. To scale impact, early integration of science communication training into curricula and dedicated funding are needed to equip plant pathologists with the tools to engage broader audiences. As part of the TRR356 we established specialized student seminars on science communication which not only generated strong interest, but also valuable media content for diverse outreach activities. Taken together, with the Botanical Garden as a neutral platform with established outreach, dedicated funding and the implementation of curricular activities, we could directly reach more than 25,000 people within less than three years of funding (excluding social media counts).

### Conclusion

Phytopathogens threaten food security, economies, and cultural heritage worldwide. Yet the success of biotechnological counter-measures hinges on societal acceptance [9]. The contrast between public trust in GM crops across regions illustrates how cultural values and institutional credibility shape adoption. Effective communication must move beyond deficit models, employing narrative storytelling, dialogue, and trusted messengers to align scientific solutions with community concerns. Botanical Gardens have a neutral reputation and attract high visitor numbers. In addition, the Botanical Gardens are highly interconnected, not only within countries but also across borders. Thus, they provide an ideal, scalable platform for outreach and education that can transform plant–microbe science into tangible, relatable knowledge and experiences [20]. The TRR356 experience demonstrates that such an approach can generate rapid, measurable engagement and plant–microbe literacy. Modular outreach elements such as exhibits, infographics, and digital add-ons can be disseminated via Botanical Garden networks to scale plant pathogen science communication.
